# MHealth approach to promote Oral HIV self-testing among men who have sex with men in China: a qualitative description

**DOI:** 10.1186/s12889-018-6046-9

**Published:** 2018-09-27

**Authors:** Yue Zhao, Xiaofang Zhu, Ashley E. Pérez, Wenhan Zhang, Anxia Shi, Zhihua Zhang, Pan Gao, Jun Wang, Cui Yang, Nickolas Zaller, Yehuan Sun, Don Operario, Hongbo Zhang

**Affiliations:** 1 School of Public Health, Anhui Medical University, 81 Meishan Road, Hefei, China; 2 Department of Social and Behavioral Sciences, University of California, San Francisco, CA USA; 3 Department of Health, Behavior and Society, Johns Hopkins Bloomberg School of Public Health, Baltimore, MD USA; 4 Health Behavior and Health Education, University of Arkansas for Medical Sciences, Little Rock, AR USA

**Keywords:** MSM, Oral HIV self-testing (oral HIVST), Mobile health (mHealth), WeChat, HIV prevention, China

## Abstract

**Background:**

HIV self-testing (HIVST) has demonstrated potential to expand HIV testing among key populations, including men who have sex with men (MSM) in China who have low testing rates. However, due to the autonomous nature of self-testing, people who undergo HIVST may lack access to relevant information and counseling support typically provided by in-person HIV testing counselors. WeChat, a popular smartphone application in China, offers a potential source of mobile health (mHealth) information and support for individuals using HIVST. This paper aimed to explore the opinions of MSM in China about the feasibility and potential concerns of using WeChat to support HIVST and reduce risk for HIV infection.

**Methods:**

We conducted in-depth interviews with 36 MSM about their mobile smartphone usage and the use of WeChat for helping MSM self-administer HIVST kits in Hefei, China. Each interview was digitally recorded and transcribed. Transcripts were analyzed used content analysis method according to Elo and Kyngas.

**Results:**

MSM described their use of WeChat and expressed cautious endorsement about using this platform to promote HIVST and disseminate HIV-related information. They described their preferences about the implementation features of an mHealth intervention to promote HIVST, including the delivery source of intervention messages, as well as message timing, frequency, form, tone, and content. Participants also described privacy-related concerns about receiving messages via WeChat and offered potential solutions.

**Conclusions:**

Findings from this study show the potential utility of WeChat app-based messaging for engaging MSM in HIV self-testing and prevention. Future research is needed to integrate the concerns expressed in this analysis and evaluate a WeChat-based intervention to promote oral HIV self-testing, risk reduction, and health promotion among MSM in China.

## Background

HIV self-testing (HIVST) is an acceptable, private, and convenient approach to HIV testing that may be beneficial to individuals who have not tested previously [1] or who prefer not to use in-person, facility-based HIV testing [2, 3]. Therefore, HIVST may be a potential strategy to increase access to HIV testing for key populations such as men who have sex with men (MSM) in many parts of the world who avoid in-person HIV testing sites due to stigma and perceived challenges to confidentiality [4, 5]. HIVST is approved by the China Food and Drug Administration (CFDA) and easily available through e-commerce websites without national policy regulation [6]. There are two kinds of HIVST: oral fluid-based HIVST and fingerstick/whole blood-based HIVST. Studies with MSM have reported preference for oral-based HIVST over fingerstick/whole blood-based HIVST [7, 8] because the former is perceived to be easier to perform and less painful [9]. A literature review concluded that the main benefits of oral fluid-based HIVST for MSM include: convenience, privacy, absence of pain, and ease of use. [9] However, reports suggest that the absence of an in-person HIV test counselor is one of the main concerns among MSM who self-administer HIVST kits in private [10, 11].

Given the increasing use of smartphones worldwide, mobile health (mHealth) interventions can improve access to public health prevention efforts in underserved communities and could make healthcare service utilization more convenient [12–14]. mHealth interventions can be particularly useful in China, where smartphone ownership is among the highest in the world [15, 16]. In particular, mHealth could be an innovative strategy to meeting HIV prevention needs in China, such as increasing HIV testing rates and disseminating HIV prevention and treatment interventions to high-risk populations such as MSM [17–19]. mHealth strategies - including text messaging or SMS [20, 21], software applications such as Facebook [22, 23], and multiple media interventions [24] - have been demonstrated as feasible for reaching community samples of MSM. However, to maximize uptake and scalability of mHealth strategies, interventions must engage the prospective end-users in order to consider the trends, patterns, and normative preferences of technology use in specific geographic settings [25].

WeChat, a mobile application released in 2011, has rapidly become one of the most popular social media apps in China [26]. WeChat provides users with integrated features such as instant messaging and social media software, and allows for the exchange of multi-media messages (text, voice, video, and image) within one’s social network with no spatial and temporal limitations. Emerging health intervention studies in China have used WeChat to promote compliance with medical recommendations and have received positive evaluations in terms of convenience and savings in time and money [27–29]. Studies conducted in China have pilot tested mHealth interventions to demonstrate the feasibility of using social media to promote HIV self-testing with MSM, primarily through providing informational videos and basic counseling to online receipients [30–32]. However, one critique of the studies conducted to date concerns the role of MSM participants as relatively passive recipients of online information designed and delivered by external agents (i.e., HIV testing agencies or research organizations), and the need for perspectives from potential MSM users about their needs, preferences, and concerns regarding mHealth-delivered HIV prevention and testing programs.

The aim of our study was to investigate the feasibility and potential concerns of using WeChat to improve oral HIVST and promote HIV risk reduction behaviors among MSM in Hefei, China. Qualitative methods were used to gain formative insight into the personal experiences and perceptions associated with WeChat and its utility for HIV prevention and self-testing. This study can provide guidance and strategies for further mHealth research using WeChat to promote oral HIV self-testing and preventive behaviors for MSM in China.

## Methods

### Participants

We recruited participants using targeted outreach (in-person and online) and referrals by a non-government organization (NGO) that provides HIV prevention and education services to MSM in Hefei, Anhui Province, China. The study was described to participants as a one-hour in-depth interview with adult men in Hefei city to learn about their smartphone usage and HIV testing and prevention education preferences. Eligible participants: 1) were Chinese men aged 18 years or older; 2) resided in Hefei; 3) had condomless anal sex with at least one male partner in the past 6 months; 4) had a smartphone on which they could use WeChat; and 5) were HIV-negative or status unknown. Thirty-six men met inclusion criteria and enrolled. Of these, 16 were older than 30 years old, 20 were younger than 30, 25 had sex exclusively with men, and 11 had sex with men and women. And 144 MSM were screened in total before interviewing final sample size (*N* = 36).

### Procedure

Interviews were conducted in a private room in a community-based HIV prevention and testing organization that serves MSM in Hefei. Before the interview started, the staff told the participants the aim and meaning of study. All participants provided informed consent and agreed to be digitally recorded. Trained research assistants then conducted a semi-structured interview, during which they asked participants about their mobile smartphone usage and about the use of WeChat for helping MSM self-administer HIVST kits. Participants received 150 RMB (approx. $24 USD) for participating. Study procedures and interviews were conducted in Mandarin by trained interviewers who were familiar with the local dialects. Data were collected between February and May 2016. Audio and interview transcript files were stored on password-protected computers. All procedures were approved by Anhui Medical University’s Internal Review Board (protocol #2015006).

### Analysis

Interview audio files were transcribed and corrected for errors and omissions by a native Mandarin speaker. Transcripts were then translated into English. Transcripts were analyzed according to content analysis method described by Elo and Kyngas (2008) [33] and coded using MAXQDA software. In accordance with Content Analysis framework, analysis followed an inductive process through three phases: preparation, organization and reporting [34]. First, Each transcript was individually read by three research team members (all native Chinese speakers) several times to being immersed in the data, selected the unit of analysis. Then, open coding continued to formulate categories and sub-categories to describe research topic, and there was also a discussion with other research team members in China and the U.S. There were some findings that addressed our primary aim to understand the perspectives of MSM about the feasibility of promoting oral HIVST, risk reduction, and health promotion in China via WeChat. Participants’ perspectives on mHealth approaches were focused on three main areas: (i) the overall acceptability of WeChat to promote oral HIVST among MSM; (ii) specific user preferences about using WeChat for receiving health promotion videos and messages; and (iii) privacy concerns about receiving HIV-related messages via WeChat.

## Results

### Acceptability of WeChat as a platform to promote HIV self-testing

All participants described the transition from traditional text messaging (SMS) to WeChat that occurred throughout China. They said that they no longer used SMS texts for the purpose of general communication due to the inconvenience and expenses of text messaging. Although few participants had ever sent or received text messages to communicate about their health with a professional counselor, those who had done so expressed several concerns about this mode of mobile communication:

*I used to consult doctors in CDC in Hefei and my hometown about the risk of getting infected by sexually transmitted diseases or AIDS and about the prevention of the infection... I think the telephone number would be revealed when sending SMS. Because the telephone number uses a real-name system now, I think the privacy cannot be protected. I mainly worried about privacy.*

Whereas use of SMS text messaging has declined for general purposes, most participants reported regular use of WeChat and said that they took advantage of the numerous features of the app in daily communication. They described using the WeChat app for a range of personal communication needs including text-based messages, voice messages, and voice calls for both personal and work purposes, and considered SMS text messaging as outdated mode of communication.

*I think the biggest advantage of WeChat is that I could send voice messages to my friends instead of text messages, after all people in the middle ages commonly don’t like typing.*

Participants reported strong interest and use of the “Moments” feature of the app, which allows users to share their ongoing thoughts and activities, such as pictures and statuses, with their social network and comment on others’ postings. Features of the WeChat app enabled participants to maintain multiple social networks with family, friends and peers, and community affiliations.

*I have a family group, some friend groups, Qinghui group (a group for gay) and Youth Health group. As for the official accounts, I have followed such accounts like environment related, scenery related, and literary related ones as well as public interests related ones.*

A few participants also described following health-related official accounts, although few reported having used WeChat for communication with health service staff. Those who had used WeChat for health communication voiced their approval of its privacy and safety. For example, a participant described the convenience in communicating with a heath provider via WeChat:

*As long as workers are professional and authoritative, I think there is no inconvenience. They were enthusiastic and gave me answers patiently. We don’t need contact with them directly or to meet face-to-face. In addition, we could consult with them whenever possible.*

All participants believed it would be acceptable to create an information platform on WeChat to support the use of oral HIVST. A few participants proposed that “Blued” (a popular gay dating application in China) could provide targeted opportunities for reaching high-risk MSM, which can complement WeChat’s broad user appeal and multi-communication features. Moreover, because of the mass use of WeChat and the millions of people in China who can potentially create topical WeChat discussion groups, participants recommended the creation of an “official” WeChat account for promoting HIVST – in which individuals would be required to request access and be invited to join – to ensure that the health information was delivered by professionals with appropriate expertise, rather than by lay community members. Creation of an official WeChat account for HIVST promotion and education would also allow for more privacy and protect against disclosure of users’ personal identities, which could be possible if accounts were maintained by private lay individuals.

*It’s better to open an official account because it is easy to reveal private information in groups. People can see each other in the groups and maybe they know each other. Because some people just don’t want others to know something about them. If you want to protect our privacy, you can open an official account. If you want to push information frequently, then you can set up a group. And some people join the group just for fun, so I think it’s better to open an official account.*

Participants expressed concern that an official service account would cause them to receive too many regular message notifications (which might automatically appear on their smart phone screen unless that feature is disabled), which might compromise privacy or inadvertently disclose their MSM behavior. They preferred a subscription account that limits the amount of “push message” notifications, and recommended that these notifications appear only when they manually enter the WeChat application.

### Specific user preferences for WeChat message development

We identified five domains described by participants as key considerations for implementation of WeChat interventions for HIVST promotion among MSM: message source, dosage, medium, tone, and content.

Due to the volume of information circulating on social media and mobile apps such as WeChat, participants primarily emphasized the credibility of the message source in order to enhance receptivity to mHealth messages. Participants perceived volunteers of recognized community-based organizations as highly credible sources of HIV and HIVST information delivered via WeChat. In particular, MSM-affiliated organizations were viewed as trustworthy sources of sexual health information. Participants described greater comfort engaging in two-way WeChat communication about sexual health with volunteers of these organizations compared with doctors, and the doctors were perceived as being insensitive and judgmental toward MSM. Participants further noted that endorsements by the Chinese Centers for Disease Control and Prevention (CDC) of WeChat accounts would confer scientific credibility alongside community credibility, thereby enhancing receptivity and trust of mHealth messages among MSM:

*Because they [volunteers] are more intimate with us. Many members among them are our peers, it will be easier for us to communicate with each other. As for the doctors, there do exist some communication barriers.*

*It is better to emphasize that this account is run by homosexual groups, so people would think their privacy will be protected. Moreover, you can tell the public that this official account is under the support of CDC, so people would think this official account is professional.*

The second key domain was message dosage, which included both frequency of WeChat messaging as well as the time of day during which messages are delivered. Most participants preferred to receive messages during non-working leisure times, and expressed caution that messages delivered during work time would be ignored. Messages delivered just prior to or after the end of the work day could maximize receipt by individuals commuting on public transportation to work. With regard to message frequency, most participants recommended sending messages two to three times a week – which was considered a balance between daily messages (perceived as too frequent and could lead to unsubscribing from the account) and weekly messages (perceived as too infrequent and could lead to concern that the account was no longer active).

Third, participants noted the need to balance across multiple types of WeChat media (text, graphic, video) to maximize interest among MSM. In order to facilitate interest in reading the WeChat messages, participants recommended the combination of text and pictures, especially cartoons/comics, to prevent them from becoming too text-heavy. Moreover, they urged that data on HIV and related health outcomes be presented in the form of simple histograms and pie charts, rather than as text. Participants expressed mixed opinions about video messages. Although participants suggested that well-produced, brief videos could effectively provide information about HIV and HIVST (e.g., demonstrations on how to self-administer HIVST kits, or videos about HIV risk in the local community), they also warned that video viewing typically requires Wi-Fi access which cannot be guaranteed at all locations. Furthermore, because video messages might openly address HIV testing and sexual behavior, participants indicated that these videos could potentially disclose their MSM behavior to people in nearby viewing distance.

*For me, I think it would be better to use pictures and text. Because I often stay in my home and it is not good for me to watch the video when my family stay around me. And it would spend much traffic if I watch it without Wi-Fi and I think pictures are more clear for me.*

The fourth key domain was message tone. Participants had previously encountered different tones by which app-based messages were delivered, ranging from formal tones, casual/friendly tones, and authoritative tones. In general, they recommended using attractive and accessible titles/content to gain people’s interest in viewing mHealth messages, especially targeted toward young people who often have high risk but little knowledge about HIV. However, participants believed that the most appropriate tone depended on the message topic; tones should vary depending on message content. For example, professional and authoritative messages may be better suited to deliver information about HIV-related medical referrals, whereas casual tones could be effective at communicating social norms about condom usage and behavioral risk reduction. Participants also recommended that messages should use simple, non-technical language to facilitate understanding, especially given the various educational backgrounds of potential recipients. For example, one participant who followed an official account specialized in disease research described that the content was difficult for him to understand due to the use of technical language. Additionally, participants agreed that the messages should avoid “preaching” and sensationalized stories, and cautioned about the use of scare tactics. They recommended that messages should be positively framed and encouraging.

Finally, participants suggested mHealth messages should present original content relevant to the MSM community, rather than repeat generic content from other mobile apps or information already available online.

*I used to follow an official account for a week to find if it is something I like, then I will decide whether to follow or to cancel the following. Some accounts are really useful, so I will read them often. If it is just trying to attract people by its headlines, but the contents are hollow/useless, I will not follow anymore.*

*If the content is lively or novel, which could attract people’s attention, I think they will accept it even if you push information every day.*

In order to reduce sexual risk behaviors and increase rates of HIV testing among MSM, participants suggested including educational messages about accurate and accessible information about the MSM-specific routes of HIV infection, high-risk behaviors that lead to the transmission of HIV, and evidence-based methods of HIV prevention in the MSM community. They also encouraged communicating local HIV epidemiological data in an accessible manner in order to raise recipients’ awareness of geographic distribution of HIV and immediate risks to themselves and their MSM peers.

*In fact, for today’s China or Anhui Province, we are not well aware of the risk of infection and infection status. We only know that this thing might exist. For many people, this thing seems to be very far, you may not be aware that there is much risk around us. So I think, for many people, raising the awareness of vigilant and encouraging people to keep fixed sexual partner can ensure that everyone keeps a relatively safe, mature attitude to face these things.*

Moreover, some believed that local HIV data was not adequately accessible to the public:

*There is no official report of current infection condition. We don’t know which organization is responsible for this. For example, how many people were infected with HIV every month in 2015 in Hefei? Or how many people were sick and the proportion of people who died? We don’t know about this kind of information, and there is no specific publishing channel. Besides, we can’t search this data on the CDC website. For people in our gay community, they hope to access this information when they need it. Although I didn’t search this data, I hope this kind of data can be published like the weather forecast, so I can access to it when I need it.*

A common recommendation about message content was to share first-person experiences from Chinese MSM living with HIV in order to personalize the epidemic.

*I think the personal experience of HIV-infected [people] can touch people’s heart, or can frighten people.*

Furthermore, to encourage HIV testing, they noted the need to include information about HIV testing times, locations, and a comparison of testing methods, and some suggested sending periodic testing reminders. Creation of a brief video of HIVST self-administration by a MSM peer was recommended to demonstrate the relative ease of use.

*If you push a video in the App or WeChat official accounts to teach others how to take self-test and let people see it, I think many people will feel that taking a test is not so trouble. Some people don’t know how to take oral test on their own.*

### Privacy concerns about HIV-related messages via WeChat

Participants expressed privacy and/or security concerns about receiving HIV-related messages via the app. Most worried that their families and friends might see the messages, which could reveal their MSM behavior. They cautioned that explicit message titles, sensitive words, and HIV-related pictures would increase the possibility of inadvertent disclosure:

*Its titles, slogans, and contents should not be too direct. It must not be recognized as HIV test related when seen by someone who isn’t a gay. If I follow it and there are some words like AIDS Prevention Office, it isn’t acceptable to me, because if it’s seen by someone else he’ll feel weird and think why I’m following such an official account.*

*Being a gay, I like to know more information about HIV transmission routes. But others may not be familiar with this disease. So if they see us viewing this kind of information by accident, they may think that I am infected.*

Participants recommended safeguards to mitigate these possibilities, including disabling the appearance of messages on the phone’s main screen, using discrete languages and imagery, and avoiding explicit MSM and HIV headlines and message titles by using colloquial language and euphemisms known by MSM.

*Because a cellphone is private, but it could be seen by many people. I hope you do not call HIV straightforwardly, but you can call it in a euphemistic way, like “red ribbon.”*

Some participants reported having two WeChat accounts – one for general communication with family and friends, and one for MSM- or HIV-specific communication – to minimize risk of inadvertent disclosure. They described this as a possible strategy to alleviate users’ privacy-related concerns:

*I have no worry about that because I have two WeChat IDs, and one of them is specifically used to focus on information related to HIV. So I have no worry about that. But I think for those people who have only one WeChat ID to receive the related information, it might better to avoid some sensitive words in the texts.*

## Discussion

Our findings indicate cautious endorsement regarding the acceptability of using WeChat to deliver messages about HIVST promotion, risk reduction, and health promotion to MSM in China. Due to the novelty of HIVST in China, WeChat offers an opportunity to disseminate information about the accuracy, safety, and appropriate use of this emerging testing method. The utility of WeChat is especially timely given the rise of mHealth interventions for HIV prevention [19, 35, 36] and testing [19] in other geographic contexts and in China [30–32], as well as the decline in use of SMS texting as a preferred mode of communication in China [37]. Compared with SMS, WeChat offers a free communication platform with multi-model (visual, text, auditory) channels that can increase engagement with health informational messages, thereby providing more complex and multifaceted information that users expect in modern communication.

WeChat can provide access to real-time information and two-way communication among MSM using HIVST in China. Due to the autonomous nature of HIVST, in which testers administer and interpret their HIV results in a private location in the absence of a trained counselor, WeChat allows access to necessary systems of support and referrals. For example, MSM who self-administer HIVST at home or other private locations can use WeChat to seek support in interpreting their HIV results, obtaining information about HIV risk-reduction strategies, and – among those who test positive – getting referrals to confirmatory testing and HIV care. Given the necessity for confirmatory testing and linkage to care for those with preliminary HIVST positive results, and for ongoing HIV risk reduction and testing behaviors for those with HIVST negative results, this type of user engagement is an important part of the self-testing experience.

Findings provide insight into ongoing concerns and strategies to optimize implementation of a WeChat system for HIVST promotion. One primary recommendation was for the creation of official WeChat accounts related to HIVST, with the accounts linked to recognized non-governmental organizations that work on behalf of MSM. Implementing this recommendation can enhance trust among MSM users, who might have fears concerning confidentiality and accuracy of HIV testing information disseminated via WeChat. Endorsement by the Chinese CDC of official WeChat accounts can further improve credibility and trust in the quality of the information. An additional concern was related to the inadvertent disclosure of MSM status or HIV risk, due to graphic content displayed on phone screens. Thus, WeChat programs for promoting and supporting HIVST should strike a balance between community-relevant content and individualized privacy in order to allay concerns about WeChat messages seen by other nearby viewers. App-based protections can include instructions to enable phone privacy features, warnings to watch graphic videos in private, and use of colloquial expressions referring to homosexuality and same-sex behavior.

Other key domains of WeChat messaging for HIVST promotion included message content, tone, and dosage. Because HIVST is a complex, multi-stage process, WeChat content can cover the steps involved in self-administering the test kit – including obtaining the oral swab sample, interpreting results, and safely disposing of testing materials – as well as advice for users in the case of a preliminary HIV–positive or negative test result. Indeed, WeChat content can convey to users that obtaining a preliminary HIV-positive result should be followed by a confirmatory test at a local CDC office. Providing access to a remote counselor via the WeChat official HIVST account can help testers who prefer to talk to a trained professional. In addition to provide information about the use of HIVST kits, WeChat messaging should include locally relevant information about the risk and mechanisms for HIV transmission among MSM, the need for regular HIV testing, options for obtaining HIV in-person testing, and the benefits of HIVST as a way to obtain preliminary HIV test results for those who experience anxiety about in-person testing. Use of WeChat multi-media such as text, short videos, graphics, and illustrations can enhance audience engagement. Messages should strike a balance of being informationally accurate and engaging to the population, and should never be judgmental about MSM behaviors and identities. The WeChat app allows for basic information to be continually accessible through a series of menus, as well as for messages to be occasionally “pushed” to users. Regarding the latter, users can optimally decide on the timing and frequency by which they receive messages based on their needs and preferences.

### Limitations

Limitations to this research must be considered. Due to the specific study site, sampling, and sample size, generalizability of the results to other settings in China or outside of China may be limited. The qualitative approach to data collection provided insight into subjective and normative perceptions about app-based HIVST messaging and support, but assessment of actual receptivity and uptake of WeChat messages and impact on HIVST behaviors requires implementation and evaluation of a WeChat intervention program. Finally, due to the ever-changing nature of mobile technology and user expectations in app-based communications, the specific recommendations reported here about WeChat as a platform for HIVST promotion and self-administration may have time-limited relevance.

## Conclusions

The current study builds on emerging evidence about the utility of WeChat and other mHealth interventions for promoting HIVST with MSM. In order to maximize the uptake, response, and effectiveness of app-delivered programs such as this, it is necessary to engage the viewpoints of end-users such as MSM to understand their preferences, needs, and concerns, and to use findings in generating subsequent generations of mHealth interventions. This research indicates cautious endorsement of WeChat as an adjunct to the future roll-out of HIVST in China. WeChat can provide a platform for accurate and sensitive information to users before, during, and after they self-administer the kit, and it can offer access to real-time counselors to answer questions and provide referrals. Ongoing development and use of WeChat interventions for HIV prevention and health promotion with MSM in China can help to engage these men in health promotion information and services. While WeChat as a platform has potential advantages to promote HST and provide health information, critical measures are needed to ensure privacy, confidentiality and to prevent unintended disclosure among MSM. Continued user feedback will be necessary to ensure that WeChat messaging is perceived as trustworthy, accurate, relevant, and confidential to this high stigmatized population.

## Abbreviations

HIVST: HIV self-testing
mHealth: mobile health
MSM: men who have sex with men
